# Gut Microbial and Metabolic Responses to Salmonella enterica Serovar Typhimurium and Candida albicans

**DOI:** 10.1128/mBio.02032-18

**Published:** 2018-11-06

**Authors:** Jennifer R. Bratburd, Caitlin Keller, Eugenio Vivas, Erin Gemperline, Lingjun Li, Federico E. Rey, Cameron R. Currie

**Affiliations:** a Department of Bacteriology, University of Wisconsin, Madison, Wisconsin, USA; b Department of Chemistry, University of Wisconsin, Madison, Wisconsin, USA; c School of Pharmacy, University of Wisconsin, Madison, Wisconsin, USA; New York University; University of California, Riverside; University of California, Irvine

**Keywords:** *Salmonella*, gut microbiome, metabolomics, metagenomics

## Abstract

The gut microbiota confers resistance to pathogens of the intestinal ecosystem, yet the dynamics of pathogen-microbiome interactions and the metabolites involved in this process remain largely unknown. Here, we use gnotobiotic mice infected with the virulent pathogen Salmonella enterica serovar Typhimurium or the opportunistic pathogen Candida albicans in combination with metagenomics and discovery metabolomics to identify changes in the community and metabolome during infection. To isolate the role of the microbiota in response to pathogens, we compared mice monocolonized with the pathogen, uninfected mice “humanized” with a synthetic human microbiome, or infected humanized mice. In *Salmonella*-infected mice, by 3 days into infection, microbiome community structure and function changed substantially, with a rise in *Enterobacteriaceae* strains and a reduction in biosynthetic gene cluster potential. In contrast, *Candida*-infected mice had few microbiome changes. The LC-MS metabolomic fingerprint of the cecum differed between mice monocolonized with either pathogen and humanized infected mice. Specifically, we identified an increase in glutathione disulfide, glutathione cysteine disulfide, inosine 5’-monophosphate, and hydroxybutyrylcarnitine in mice infected with *Salmonella* in contrast to uninfected mice and mice monocolonized with *Salmonella*. These metabolites potentially play a role in pathogen-induced oxidative stress. These results provide insight into how the microbiota community members interact with each other and with pathogens on a metabolic level.

## Abstract (precis)

The gut microbiota is increasingly recognized for playing a critical role in human health and disease, especially in conferring resistance to both virulent pathogens such as *Salmonella*, which infects 1.2 million people in the United States every year (E. Scallan, R. M. Hoekstra, F. J. Angulo, R. V. Tauxe, et al., Emerg Infect Dis 17:7–15, 2011, https://doi.org/10.3201/eid1701.P11101), and opportunistic pathogens like *Candida*, which causes an estimated 46,000 cases of invasive candidiasis each year in the United States (Centers for Disease Control and Prevention, *Antibiotic Resistance Threats in the United State*s, *2013*, 2013). Using a gnotobiotic mouse model, we investigate potential changes in gut microbial community structure and function during infection using metagenomics and metabolomics. We observe that changes in the community and in biosynthetic gene cluster potential occur within 3 days for the virulent Salmonella enterica serovar Typhimurium, but there are minimal changes with a poorly colonizing Candida albicans. In addition, the metabolome shifts depending on infection status, including changes in glutathione metabolites in response to *Salmonell*a infection, potentially in response to host oxidative stress.

## INTRODUCTION

Symbiotic microbes help shape the biology of plants and animals (1). In humans, gut microbes modulate nutrition and immune function and are correlated with an increasing number of metabolic and neurological health and disease states (2, 3). The human gastrointestinal tract harbors the largest fraction of microbial life in the body, estimated to range from 10^8^ to 10^10^ bacteria per gram in the ileum and stool, respectively (4). Bacteria are the dominant taxa in the human gut microbiome, with the most abundant lineages belonging to the phyla *Bacteroidetes* and *Firmicutes*. Nevertheless, these communities are highly diverse and include viruses, archaea, fungi, and protists (5–8), and all combined contain 150 times as many genes as the human genome (9). In a healthy state, the human gut microbiome is relatively stable over time (10, 11). Major disruption of the gut microbiome is associated with infections by a number of serious human pathogens, such as Clostridium difficile, vancomycin-resistant *Enterococcus* (VRE), and Salmonella enterica (12–14).

Preventing exogenous microbes from colonizing the human intestine is critical to the host maintaining a stable and healthy gut microbiome. The role of the microbiome in preventing pathogens from invading the gut has been recognized since the 1950s, when pretreatment with antibiotics was shown to drop the infectious dose of Salmonella enterica 100,000-fold (15). Gut microbes confer colonization resistance by outcompeting pathogens for nutrients, priming the host immune system, and directly targeting other microbes with metabolites (16). Several examples of metabolites produced or modified by the microbiota that inhibit pathogens include short-chain fatty acids, secondary bile acids, and modified compounds from the diet (17–19). In addition, some members of the microbiota can create compounds to respond selectively to pathogen infection (20). The gut microbiota has the potential to make a wide variety of novel natural products, and many of the large biosynthetic gene clusters encoding natural products are found in relatively small genomes, indicative of an ecological role for the products (21).

Experiments using gnotobiotic mice with and without human microbiota, in combination with metagenomic and metabolomic approaches, can provide insight on the structure and function of the gut microbiota during pathogen invasion. Gnotobiotic mice are a mammalian model system in which defined microbiomes can be used in a controlled environment. Various metabolomics techniques, including nuclear magnetic resonance and chromatography-mass spectrometry, have been used for large-scale characterization of metabolite changes as a result of microbiome colonization, illustrating the impact of the microbiota on not only intestinal metabolism but also global systems (22, 23). Furthermore, liquid chromatography-mass spectrometry (LC-MS) can help to characterize metabolite changes due to disturbances in the microbiome (24, 25) and to screen for novel secondary metabolites and natural products in bacterial systems (26, 27).

Here we examine colonization resistance in the humanized (HUM) mouse model. Specifically, we perform experimental infection with Salmonella enterica serovar Typhimurium and Candida albicans in HUM mice and in germfree (GF) mice. Salmonella enterica Typhimurium is a disruptive pathogen that causes massive inflammation to outcompete the native microbiota in mice and human models (28–30). Candida albicans can cause low-grade inflammation, but in contrast to Salmonella enterica Typhimurium is considered a commensal and occasional opportunistic pathogen in the GI tract (31–34). Nevertheless, C. albicans has been shown to colonize GF and antibiotic-treated adult mice (33, 35, 36), which appear otherwise resistant, suggesting that gut microbiota play a role in preventing *Candida* colonization in mice and humans. In this study, we investigate how these pathogens alter the structure of the human gut microbiome, the biosynthetic gene cluster potential, and the metabolites produced in a healthy or infected state. We cross the presence and absence of the microbiome with the presence and absence of pathogen infection, using either S. enterica Typhimurium or C. albicans. To characterize strain-level diversity that is not resolvable with 16S rRNA gene sequencing, we use shotgun metagenomics on fecal samples over 3 days of infection. We also identify the capacity of community members to produce novel antimicrobials through the biosynthetic gene clusters embedded in bacterial genomes. Further, we characterize metabolites using LC-MS for relative quantification and discovery metabolomics in the host cecum during infection and validate the identifications of several specific metabolites with commercial standards.

## RESULTS

### Infection severity in mice with and without microbiota.

**FIG 1:**
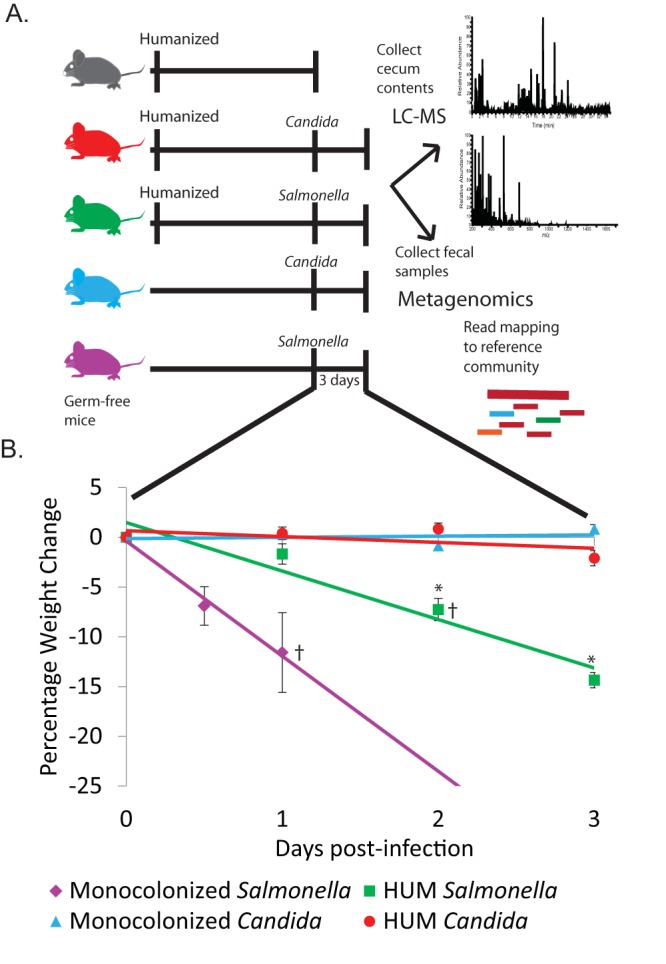
(A) Overview of experimental design. (B) Percent body weight loss during 3 days of infection. Errors bars indicate standard error. Significant difference from HUM *Candida* (*P* < 0.05) using Wilcoxon test denoted by * next to relevant group. Mice sacrificed early indicated with † (monocolonized *Salmonella*, 3 at 12 h and 3 at 24 h, HUM *Salmonella* 1 at 24 h).

Germfree mice, 8 to 12 weeks old, were kept germfree or colonized via oral gavage with a synthetic human community for 2 weeks, and then infected with Salmonella enterica Typhimurium or Candida albicans (Fig. 1A). All infected mice showed presence of pathogens in fecal samples by growth on selective media. Prior to infection, the mice weighed on average 29.8 g ± 2.3 (mean ± SD). GF mice infected with *Salmonella* (*n* = 6), henceforth referred to as monocolonized *Salmonella* mice, lost an average body mass of 2.0 ± 1.4 g or 6.8% ± 4.7% within 12 h postinfection. Due to severity of symptoms, three monocolonized *Salmonella* mice were sacrificed 12 h postinfection, and the remaining monocolonized *Salmonella* mice and one HUM mouse infected with *Salmonella* were sacrificed within 24 h of infection. HUM mice infected with *Salmonella* surviving 3 days into infection (*n* = 5) lost an average of 4.2 ± 0.6 g or 14.3% ± 1.7%, a significant loss in comparison to weight change from both the monocolonized and HUM mice infected with *Candida* (Mann-Whitney U test, Bonferroni corrected, *P* < 0.05). The monocolonized *Candida* mice (*n* = 6) gained on average 0.2 ± 0.3 g or 0.8% ± 1.1% of their original weight, and the *Candida-*infected HUM mice (*n* = 6) gained on average 0.7 ± 0.5 g or 2.0% ± 1.8% of their original weight. There was no statistically significant difference in the change in weight for the monocolonized *Candida* mice compared to the HUM mice infected with *Candida* by the endpoint of the experiment, 3 days of infection.

### Microbial community shifts in response to infection.

**FIG 2:**
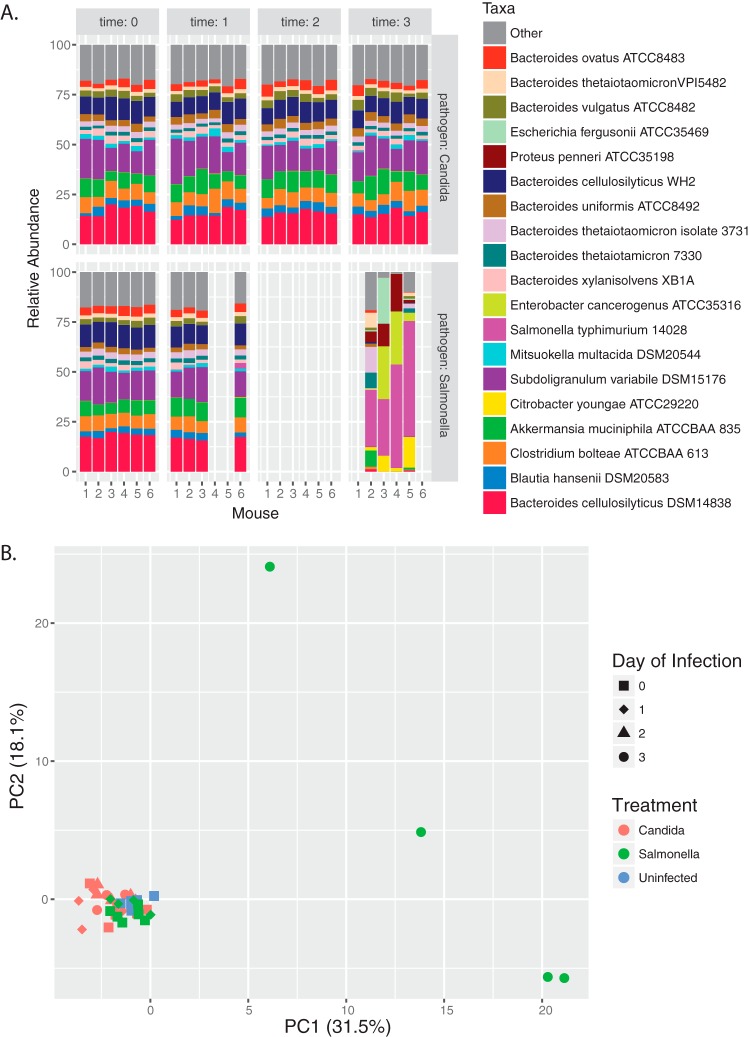
Variation in fecal microbiota metagenomes during infection. (A) Relative abundance of top 19 strains in HUM Candida albicans and Salmonella enterica Typhimurium infection group. (B) PCA of strain relative abundance.

We conducted Illumina-based metagenomic sequencing on DNA from fecal pellets collected throughout infection. Each sample had on average 407,535 reads (SD = 63,381), ranging from 295,235 to 523,271 reads. The average number of reads with at least one reported alignment was 385,882 ± 96,477, or 95% of reads per sample. Prior to infection, the most abundant strains, making up over half of the relative abundance in the metagenomes from all groups, were Bacteroides cellulosilyticus DSM14838, Subdoligranulum variabile, Bacteroides cellulosilyticus WH2, Akkermansia muciniphila, and Clostridium bolteae with an average relative abundance of 15.1%, 14.1%, 9.1%, 7.8%, and 6.5%, respectively (Fig. 2A). By day three in the *Salmonella-*infected HUM mice, most of the communities were dominated by *Salmonella* and other various *Enterobacteriaceae* strains from the original inoculum. Furthermore, diversity significantly decreased in *Salmonella*-infected mice (see Fig. S1 in the supplemental material). Prior to infection, these strains (C. youngae, P. penneri, E. cancerogenus, and E. fergusonii) in total represented an average relative abundance of 0.2%. In the metagenomes from two mice, we observed an increase in the reads mapping to Enterobacter cancerogenus, up to 26.4% and 26.6% of the community, along with a smaller increase in Proteus penneri. One mouse had an increase in Escherichia fergusonii to 22.9% of the metagenome, while it remained below 1% of the metagenome in all the other mice. In another mouse, Citrobacter youngae reads increased to 15.2%, while in other mice C. youngae reads remained below 7.9%. After excluding *Salmonella* reads, we continued to observe a large shift in the relative abundance of community members. Using principal component analysis (PCA), we show large separation of the HUM *Salmonella* microbiome communities, 3 days postinfection, from a tight cluster of all other time points and treatments, with the first component explaining 31.4% of the variation (Fig. 2B).

10.1128/mBio.02032-18.1 FIG S1 Community diversity before and 3 days postinfection. Download FIG S1, EPS file, 0.8 MB. Copyright © 2018 Bratburd et al. 2018 Bratburd et al. This content is distributed under the terms of the Creative Commons Attribution 4.0 International license.

In all *Candida*-infected HUM mice, less than 1% of reads mapped to the Candida albicans SC5314 reference genome. The metagenome of this group was not significantly different from uninfected HUM mice. The community structure remained fairly consistent over the infection period, although there was some variation in strain relative abundance over time (Fig. 2). The largest change in any individual strain’s relative abundance was an 8.4% increase in Subdoligranulum variabile in one mouse from 1 day postinfection to 3 days postinfection.

### Prevalence of biosynthetic gene clusters within genomes and metagenomes.

In total, from the genomes of the human microbiome used in this study, using antiSMASH 4.0 (37), we detected 1,081 biosynthetic gene clusters (BGCs). Of these clusters, when grouped together using BiG-SCAPE with a cutoff distance of 30 calculated based on a weighted combination of Jaccard, domain sequence similarity, and adjacency index, we identified 128 cluster nodes in 51 groups. The remaining 953 BGCs did not form any groupings with each other. Based on antiSMASH-predicted classifications, most clusters were classified as other, which included putative clusters (486), fatty acids (117), fatty acid-saccharide combined clusters (22), aryl polyenes (14), siderophores (4), and resorcinol (3). Another large category was saccharides (345), followed by 62 ribosomally synthesized and posttranslationally modified peptides (RiPPs), a group that includes bacteriocins, sactipeptides, lantipeptides, and thiopeptides. We also found 20 nonribosomally synthesized peptide clusters and one hybrid polyketide-NRPS cluster in Desulfovibrio piger (Table S1).

10.1128/mBio.02032-18.5 TABLE S1 Biosynthetic gene clusters predicted in humanized microbiota. Download Table S1, DOCX file, 0.01 MB. Copyright © 2018 Bratburd et al. 2018 Bratburd et al. This content is distributed under the terms of the Creative Commons Attribution 4.0 International license.

**FIG 3:**
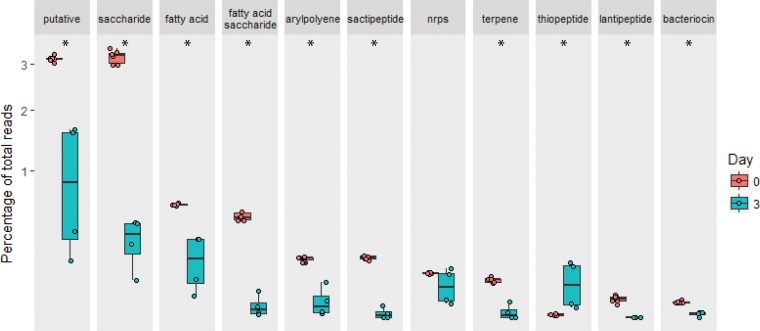
Percent abundance of reads mapping to biosynthetic gene clusters out of total reads that were mapped from the metagenome from HUM *Salmonella*-infected mice prior to infection (*n* = 6) and 3 days into infection (*n* = 4), on a square root-adjusted axis. Significance (*P* < 0.05 with Benjamini-Hochberg correction) is indicated with *.

We found significant differences in the percentages of total metagenomic reads mapping to BGCs in *Salmonella*-infected HUM mice prior to infection versus 3 days postinfection (Wilcoxon *P* < 0.05, corrected with Benjamini-Hochberg), excluding reads mapping to BGCs from *Salmonella* itself. Saccharides, lantipeptides, aryl polyenes, sactipeptides, fatty acids, fatty acid-saccharides, terpenes, and putative clusters were significantly reduced, while thiopeptides significantly increased 3 days postinfection (Fig. 3). The majority of non-*Salmonella* reads mapping to thiopeptide clusters mapped to Citrobacter youngae, Enterobacter cancerogenus, Proteus penneri, and Escherichia fergusonii, consistent with the overall increase relative abundance in *Enterobacteriaceae* described above.

### Differential metabolomics during infection and novel metabolite potential.

Analysis of the LC-MS results with Compound Discoverer (Thermo Fisher Scientific) resulted in the grouping of 8,613 merged features (chromatographic peaks) into 8,259 putative compounds. The compounds detected from the cecum samples of one or more mice from each treatment group totaled 3,254 for the monocolonized *Candida* mice, 3,696 compounds for the monocolonized *Salmonella* mice, 3,349 compounds for the uninfected HUM mice, 2,924 compounds for the HUM mice infected with *Candida*, and 2,815 compounds for the HUM mice infected with *Salmonella*.

**FIG 4:**
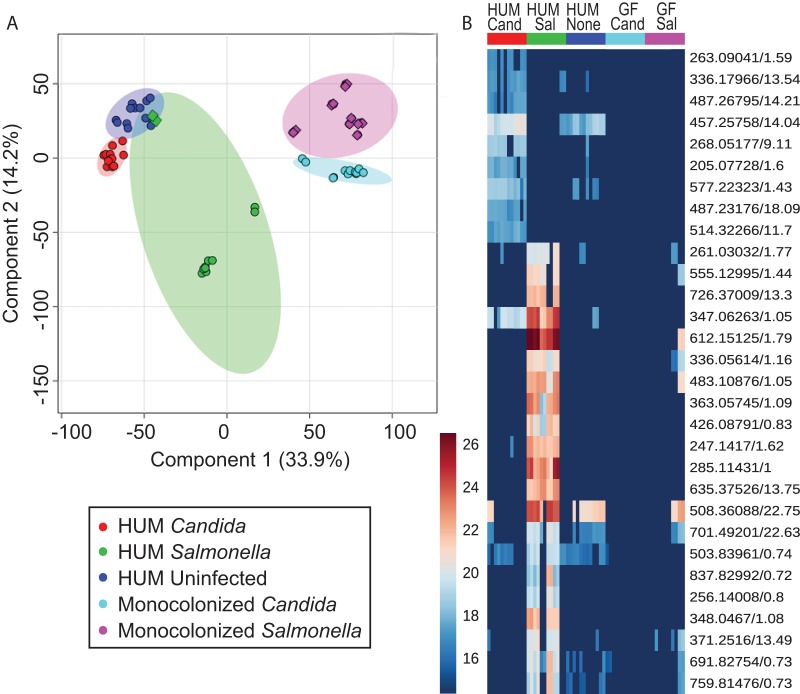
(A) PLS-DA of metabolites from all groups, with 95% confidence intervals. (B) Metabolites of interest 1.5× higher in HUM infected groups than uninfected mice, absent in 8/10 technical replicates for monocolonized mice. Circles are samples collected three days postinfection; diamonds are from animals sacrificed 1 day postinfection.

LC-MS *m/z* values and relative intensities from cecum contents showed separation of samples with PCA. Two components were able to explain 67.7% of the variance (Fig. S2). Using partial least-squares discriminant analysis (PLS-DA), we observed distinct separation of all groups with two components (R2 = 0.70799, Q2 = 0.66183 for component 1 and R2 = 0.85972 and Q2 = 0.81188 for component 2; Fig. 4A). Using permutation testing of the PLS-DA, we obtained statistical significance (*P* < 0.001) for 1,000 permutations. The outliers in the *Salmonella*-infected HUM mouse group were from two technical replicates of one sample that had to be sacrificed 24 h into infection. We also found distinct patterns for different groups of metabolites (Fig. 4B), which indicate similar patterns between uninfected HUM and *Candida*-infected HUM mice compared to monocolonized infected mice and HUM *Salmonella* mice. Additionally, we identified numerous features overrepresented in the monocolonized groups compared to the HUM groups (Fig. S3).

10.1128/mBio.02032-18.2 FIG S2 Principal component analysis of all metabolites detected. Download FIG S2, EPS file, 2.8 MB. Copyright © 2018 Bratburd et al. 2018 Bratburd et al. This content is distributed under the terms of the Creative Commons Attribution 4.0 International license.

10.1128/mBio.02032-18.3 FIG S3 Heatmap of all *m/z* detected. Download FIG S3, EPS file, 3.2 MB. Copyright © 2018 Bratburd et al. 2018 Bratburd et al. This content is distributed under the terms of the Creative Commons Attribution 4.0 International license.

To examine metabolites potentially produced by the microbiome in response to infection, we looked for metabolites that were typically not found in pathogen-monocolonized mice (absent in at least 8 of 12 samples, representing 6 biological replicates with 2 technical replicates each) and were at least 1.5-fold higher in abundance in infected HUM mice compared to the highest normalized area of the controls (HUM mice with no infection). Using these guidelines, we narrowed our metabolites of interest to 31 out of 8,085 features detected overall. We detected 22 features in higher abundance in HUM *Salmonella*-infected mice. In HUM *Candida*-infected mice, we found 10 features of interest based on the above criteria. One metabolite (*m/z* 347.0626, retention time 1.05 min) appeared to be shared between the lists, and also had matching tandem MS fragmentation from both infection groups. This metabolite had similar MS/MS to 3′AMP and 2′AMP standards, but the experimental retention time did not match that of the standards (1.37 min for 3′AMP and 2.22 min for 2′AMP). From the 31 selected compounds of interest, only 6 from HUM *Salmonella* and 4 from *Candida* infection had putative identifications based upon accurate mass matching to KEGG, HMDB, or AntiBase, leaving a remaining total of 21 potentially novel compounds (Table S2). *In silico* fragmentation with MetFrag (38) was performed using MS/MS spectra obtained on the targets. If the top peaks in the experimental MS/MS were explained by the *in silico* fragmentation, then standards were obtained to confirm the identification. Using this procedure, we identified glutathione disulfide, glutathione cysteine disulfide, inosine 5′-monophosphate, and hydroxybutyrylcarnitine as compounds upregulated from the HUM *Salmonella* group (Fig. S4). Although the *in silico* fragmentation approach worked well for the targets with KEGG matches, the increasing number of compounds in the more inclusive databases made it difficult to find putative identifications with MS/MS for targets that did not have matches to the KEGG databases.

10.1128/mBio.02032-18.4 FIG S4 MS/MS spectra of experimental compounds and matching standards. Download FIG S4, EPS file, 2.5 MB. Copyright © 2018 Bratburd et al. 2018 Bratburd et al. This content is distributed under the terms of the Creative Commons Attribution 4.0 International license.

10.1128/mBio.02032-18.6 TABLE S2 Features of interested in humanized infected mice. Download Table S2, DOCX file, 0.01 MB. Copyright © 2018 Bratburd et al. 2018 Bratburd et al. This content is distributed under the terms of the Creative Commons Attribution 4.0 International license.

## DISCUSSION

Understanding how microbial communities change in response to perturbation is crucial for health, not only because the microbiota can protect the host against pathogenic microbes but also because changes in the gut microbiota have been associated with multiple health conditions (39). Increasingly it has been recognized that pathogenicity and virulence can depend on the context of specific microbe-microbe interactions or the whole community, indicating the importance of studying pathogen-microbiome interactions (40, 41). In this study, we compare how two pathogenic perturbations affect the structure and function of human gut microbiota in a gnotobiotic mouse model. We find that during infection with *Salmonella*, the structure and functional capacity of the microbiota change. Corresponding to these changes, we see significant changes in metabolites before versus during infection that vary with and without the human microbiota.

Our infection experiments revealed significant differences among treatments as measured by weight loss. *Candida*-infected mice had weights that remained around their baseline starting weight. While we did isolate CFUs of *Candida* from mouse feces using media with antibiotics, indicating that viable yeast cells passed through the host, reads mapping to *Candida* from the metagenomic data were at or below the limit of detection, suggesting that *Candida* did not readily colonize these mice. Alternatively, the lack of fungal DNA may be influenced by our DNA extraction method (42). In contrast, *Salmonella-*infected mice lost significantly more weight than *Candida-*infected mice by 3 days into infection, regardless of microbiome presence or absence. GF mice infected with *Salmonella* were moribund within 24 h, while HUM mice infected with *Salmonella* were able to survive until the end of the 3 days, with the exception of one mouse, indicative of the protective effects of the microbiota against *Salmonella*.

*Salmonella* infection perturbed the microbiota and led to an increase in the relative abundance of different *Enterobacteriaceae*, whereas *Candida* did not. Prior to infection, the microbiota contained similar dominant taxa including *Bacteroidetes* and *Firmicutes* with relatively few *Gammaproteobacteria*. During *Salmonella* infection in humanized mice, the metagenomic data indicated an increase in the relative abundance of *Enterobacteriaceae* (including strains besides *Salmonella*). This result is consistent with previous work examining changes in gut microbial communities during *Salmonella* infection (28, 43, 44), and resembles increases in Enterobacteriaceae during antibiotic treatment (13), both of which may ultimately be driven by the oxygenation of the gut (45). These changes may represent a bloom of closely related strains or a reduction in the size of the bacterial community overall. Although Enterobacteriaceae increased in the samples, which particular strains increased appeared stochastic. Some of the variation may be due to read mapping of conserved genes to closely related strains; however, we saw similar results using different read mapping programs (Bowtie and Burrows-Wheeler Algorithm) and using parameters to exclude non-uniquely mapping reads. Given that these strains may compete with *Salmonella* over electron acceptors and trace elements, further investigation on these dynamic interactions is warranted (46, 47). The stochasticity may also reflect the general instability of the community. While *Salmonella* dramatically perturbs the community, *Candida* did not seem to readily colonize the mice, and although some changes occurred in the microbial communities, these fluctuations are within the range of natural variation.

The synthetic human microbiome used in this study contained many biosynthetic gene clusters, and the potential functional capacity changed with infection treatment. In our input strains we found potential for unknown biosynthetic gene clusters, including RiPPs, NRPS clusters, and many putative clusters. This fits with previous observations; biosynthetic gene clusters are common in human gut microbiota and anaerobic bacteria (21, 48). Metagenomic analysis indicated a decrease in most cluster types during *Salmonella* infection, which likely reflects a drop in community diversity. One exception was the increase in reads mapping to gene clusters involved in thiopeptide biosynthesis, which was increased even after removing reads mapping to *Salmonella*’s own thiopeptide biosynthesis cluster. Thiopeptides are a class of peptide antibiotics that target Gram-positive bacteria (49). Since *Salmonella* is Gram-negative and has one putative thiopeptide BGC of its own, it seems unlikely that these thiopeptide clusters, if produced, would target *Salmonella*. Other possibilities are that if produced, these secondary metabolites encoded by clusters might add to the community instability, or that these genes are not transcribed or translated. Alternatively, this result may suggest that the pathogen-induced disruption in the microbiome helps diminish members that would have been capable of producing BGC products. Further research will be needed to characterize what role, if any, these BGCs play during infection.

Our discovery metabolomics showed differences in the metabolites present in the mouse cecum based on presence of microbiome as well as infection. For example, the metabolomes of *Salmonella*-infected, *Candida*-infected, and uninfected mouse ceca grouped separately on PLS-DA analysis, suggesting distinct metabolic responses between a virulent bacterial pathogen and opportunistic fungal pathogen. The changes in overall metabolites based on gut microbiota support previous research comparing germfree and colonized mice and mice with different gut microbiome donors (50). We found more putative metabolites of interest (based on higher abundance in HUM infected mice and generally absent in GF mice) from *Salmonella*-infected mice than *Candida*-infected mice. Previous studies investigating global metabolomics in *Salmonella* infections have focused on the hosts with conventional mouse microbiota, finding disruptions in host hormone pathways (51), changes in common microbial metabolites, including trimethylamine *N*-oxide (TMAO) and hippurate (52), and changes in sugar moieties (43). Our study differed from these previous studies in that we used gnotobiotic mice to specifically focus on metabolites produced when human-associated gut microbiota strains were exposed to pathogens. While using native microbiota to look for pathogen interactions is valuable especially in an ecological context, the humanized mouse model enables exploration of potentially distinct chemical interactions between human microbiota strains and human pathogens (53). Furthermore, human gut microbiota extracts have been previously shown to inhibit virulence of *Salmonella in vitro* (40). Mice monocolonized with pathogens serve as key controls that allowed us to focus on compounds apparently made by the microbiota during infection rather than overall host changes. Nevertheless, the possibility exists that we may detect metabolites made by *Salmonella* in response to gut microbiota in our experiments or metabolites that differ due to GF mice exhibiting colitis rather than the typical systemic typhoid-like infection (54). In addition, we scanned for molecular features with an *m/z* greater than 200, to avoid discovery of smaller commonly made microbial metabolites. In our metabolites of interest from humanized infection conditions, we had many molecular features that were not identified with KEGG, HMDB, or AntiBase, potentially indicating novel metabolites. One drawback in studying these metabolite interactions *in vivo* is the challenge in isolating individual novel molecules from a complex mixture, even in a well-described community with full genomes (55), as we were unable to match known and predicted metabolites to a majority of our target *m/z* values. Although work is being done to increase MS/MS databases for natural products (56), identifying natural products is still challenging, as many natural product databases, including AntiBase, are not MS compatible.

We were able to identify a few metabolites specific to the humanized *Salmonella*-infected group, including two metabolites in the glutathione pathway. In particular, we identified glutathione disulfide and glutathione cysteine disulfide in higher abundance in humanized *Salmonella*-infected mice. *Salmonella* infection triggers vast amounts of oxidative stress (57), and glutathione metabolism is important for protection against oxidative stress (58). Changes in genes encoding antioxidant proteins have also been identified in humans exposed to Salmonella enterica serovar Typhi (59). Further, glutathione cysteine disulfide has been shown to reduce colonic lesions in a mouse model of colitis (60). Previous work indicates that germfree mice have a disrupted glutathione metabolism relative to conventional mice (61). It remains to be seen whether experimentally manipulating glutathione metabolite amounts affects *Salmonella* infections *in vivo*, and to what extent different gut microbes contribute to the glutathione pool. In contrast to the hypothesis that microbes may make specific metabolites that inhibit certain pathogens, this evidence suggests more generalized responses to certain kinds of dysbiosis, such as oxidative stress (62). The possibility of microbial metabolites with specific responses to pathogens cannot be eliminated; however, many metabolites remain unidentified, and the roles of those identified are unclear. Further characterization of microbial metabolites made during infection is necessary to identify these responses.

Colonization resistance conferred by the microbiota helps the host resist a variety of pathogens, including *Salmonella*. Understanding the complex interactions between the host, microbiota, and pathogens will enable better microbiome based-therapies, from fecal microbiota transplants to microbiota-derived compounds (63, 64). Combining gnotobiotic mice with genomics and metabolomics has allowed us to interrogate changes in community composition and function during infection in an unbiased manner and demonstrates distinct metabolic responses to a virulent or opportunistic pathogen.

## MATERIALS AND METHODS

### Human gut microbiota and pathogens.

For our synthetic human microbiome gut community, we used a collection of previously obtained isolates cultured from human fecal samples and maintained in long-term storage in the Rey lab at the University of Wisconsin-Madison. Bacterial isolates (Table S3) were grown from glycerol stock on Mega Medium (65), which was filter sterilized and held in a Coy anaerobic chamber (5% H_2_, 20% CO_2_, and 75% N_2_). An even mix from each bacterial culture was inoculated into each anaerobic tube. From stock cultures, Salmonella enterica Typhimurium ATCC 14028 was grown aerobically overnight in LB broth at 37°C, while Candida albicans K1 was grown on Sabouraud dextrose agar (SDA).

10.1128/mBio.02032-18.7 TABLE S3 Strains used in humanized community. Download Table S3, DOCX file, 0.02 MB. Copyright © 2018 Bratburd et al. 2018 Bratburd et al. This content is distributed under the terms of the Creative Commons Attribution 4.0 International license.

### Gnotobiotic mice and experimental infections.

The University of Wisconsin-Madison Animal Care and Use Committee approved protocols used in mouse experiments. GF male C57BL/6J mice were maintained in gnotobiotic isolators until 8 to 12 weeks of age with 12-h light cycle and sterilized food and water *ad libitum*. These GF mice were then randomly assigned to 1 of 5 treatment groups, moved to out-of-the-isolator gnotobiotic cages in autoclaved filter-top cages, and subsequently gavaged in a biosafety cabinet using aseptic technique (66). Mice were housed 3 per cage, with a total of 6 mice per group.

To humanize mice, GF mice were colonized via oral gavage with 0.2 ml mixed bacterial culture as shown in Table S3. All HUM mice were given the same inoculum, where bacteria were mixed with roughly similar proportions. Prior to infection, HUM mice were given 2 weeks to allow stabilization of the community. For mouse infections, mice were inoculated via oral gavage with 0.2 ml of overnight culture of Salmonella enterica Typhimurium ATCC 14028 or Candida albicans K1. Humanization and infection treatments were performed in a biosafety cabinet using aseptic technique (66). Mice were sacrificed 3 days postinfection or earlier depending on symptom severity and weight loss. Cecal contents were collected, flash frozen and stored at −80**°**C until processing. We selected cecum contents for LC-MS due to their high microbial loads and proximity to the distal ileum to which *Salmonella* localizes (28, 67).

*Salmonella* and *Candida* quantification was performed by serial dilutions of fecal samples in phosphate-buffered saline, followed by plating for quantification for *Salmonella* on xylose lysine deoxycholate (XLD) agar, and for *Candida* on SDA with chloramphenicol and gentamicin. Fecal samples from uninfected mice showed no growth on the SDA plates, as well as no growth of black colonies on XLD plates, indicating no colonies capable of metabolizing thiosulfate into hydrogen sulfide as *Salmonella* does.

### Metagenomics.

To characterize the gut microbiome of HUM mice, we conducted metagenomics using Illumina MiSeq. Genomic DNA was extracted from fecal pellets following the Turnbaugh et al. protocol (68). Briefly, the protocol is as follows: to each frozen fecal pellet, we added 500 μl of extraction buffer (200 mM Tris, 200 nM NaCl, 20 mM EDTA), 210 μl 20% SDS, 500 μl phenol-chloroform, 500 μl 0.1-mm zirconia-silica beads, and one 3.2-mm stainless steel bead. Cells were beaten for 3 min at room temperature. To remove contaminants, the Wizard SV Gel and PCR Clean-up kit was used. DNA library preparation and sequencing were done at the University of Wisconsin-Madison Biotechnology Center. Samples were prepared with the TruSeq Nano DNA LT Library Prep kit (Illumina Inc., San Diego, CA, USA) with minor modifications. After shearing samples with a Covaris M220 Ultrasonicator (Covaris Inc., Woburn, MA, USA), samples were size selected for an average insert size of 550 bp using SPRI bead-based size exclusion, and then libraries were standardized to 2 nM. Sequencing was done using single ends on the Illumina MiSeq sequencer with a 50-bp (v2) sequencing cartridge.

Metagenomic data were preprocessed using BBDuk (https://sourceforge.net/projects/bbmap/) to trim adapters, remove phi-X contamination, and quality trim reads to Q10. We analyzed the reads using the COPROseq (Community profiling by sequencing) pipeline (69), which mapped reads to reference genomes using Bowtie version 1.0 (70), and normalized reads based on genome length. We also compared read mapping using the Burrows-Wheeler Alignment tool to verify that reads mapped consistently (71). Reference genomes were obtained from NCBI. Diversity was analyzed using the vegan package in R with a Kruskal-Wallis test. Biosynthetic gene clusters were identified using antiSMASH 4.0 (37). Gene clusters were then grouped by similarity using BiG-SCAPE (J. Navarro-Muñoz et al., unpublished data; https://git.wageningenur.nl/medema-group/BiG-SCAPE). Data were analyzed and figures produced in R. Statistical testing was done using a Wilcoxon rank sum test (Mann-Whitney U test) with a Benjamini-Hochberg correction.

### Metabolomics.

All chemicals were obtained from Fisher Scientific unless otherwise noted. Mouse cecum samples were placed in 10-ml PTFE tubes for extraction with a methanol-chloroform/water extraction. Three parts methanol, 1 part chloroform, and 4 parts water (Milli-Q system, Millipore, Billerica, MA) were added, in order, to each sample (total volume, 4 ml) and centrifuged for 20 min at 4,575 × *g* at 4°C. The aqueous fraction was removed, and 4 parts methanol were then added. After brief vortexing, samples were centrifuged for 5 min at 1,500 × *g* and 4°C. The organic layer was removed. Samples were dried in a SpeedVac and stored at −80**°**C. To clean up the sample, the aqueous fraction was further processed with a 3-kDa molecular weight cutoff (MWCO) (Amicon Ultra, Millipore). The MWCO device was rinsed with 0.2 ml 0.1 M NaOH and 0.5 ml 50/50 methanol-water. The sample was loaded in 0.5 ml 50/50 methanol-water and rinsed with 0.1 ml 50/50 methanol-water. All centrifugations occurred at 14,000 × *g* until the rinse or sample was through the device. The MWCO flowthrough was dried with a SpeedVac and stored at −80**°**C until analysis.

Aqueous samples were resuspended in optima-grade water at a concentration of 10 mg/ml. A Dionex Ultimate 3000 UHPLC system (Thermo Scientific, Waltham, MA, USA) and a Cortecs C_18_ column (2.1-mm internal diameter × 100-mm length, 1.6-μm particle size; Waters, Milford, MA, USA), equipped with a corresponding guard column were used to separate the samples. The column temperature was 35°C, and the mobile phases were optima-grade water with 0.1% formic acid (A) and acetonitrile with 0.1% formic acid (B). The separation occurred with a 35-min gradient at a flow rate of 0.3 ml/minutes with the following conditions: 0 to 5 min, 1% B; 5 to 10 min, linear gradient from 1% to 3% B; 10 to 18 min, linear gradient from 3% to 40% B; 18 to 22 min, linear gradient from 40% to 80% B; 22 to 27 min, column cleaning at 95% B; and 27 to 35 min, reequilibration at 1% B. The injection volume was 3 μl and the samples were kept at 10°C during analysis. Metabolite MS data were acquired on a Q-Exactive Orbitrap mass spectrometer (Thermo Scientific, Waltham, MA, USA), which was equipped with an ESI source and operated in positive ion mode with a scan range of *m/z* 200 to 1,700. The MS parameters were as follows: 70,000 resolution, 1E6 AGC, and 100-ms maximum injection time.

### Metabolomics data analysis.

Relative quantification of the metabolomics data for the different sample types was performed with Compound Discoverer software (Thermo Scientific, Waltham, MA, USA). Spectra underwent retention time alignment (adaptive curve 5 ppm, 1-min tolerances), detection of unknown compounds (5 ppm, 30 intensity threshold, 3 S/N threshold, 1,000,000 minimum peak intensity), and grouping of unknown compounds (5 ppm, 0.05 retention time tolerance). The Compound Discoverer workflow also included fill gaps, mark background, predict compositions, ChemSpider search, normalize areas (constant sum), merge features, and differential analysis. To isolate metabolites of interest, *m/z* values detected in the blanks or in more than 4 of 12 replicates in either of the germfree infected conditions were removed. Additionally, *m/z* were selected if they showed 1.5-fold upregulation in 8 of 12 replicates of the infected humanized group, with the ratios being calculated from the control with the highest normalized area. MetaboAnalyst (72, 73) was used for further statistical analysis after exporting *m/z* values, retention time, and normalized areas from Compound Discoverer. Data were filtered with an interquantile range (IQR) estimate and log transformed. Heatmaps were produced using Pearson and Ward clustering.

### Compound identification.

MS/MS spectra for the compounds on the target lists for both infections were collected on the Dionex UltiMate 3000 UHPLC and Q-Exactive instrument described above. The injection volume was 20 μl. An inclusion list was used for the targets with a retention time window of ± 0.7 min. All charge states and salt adducts observed in the Compound Discoverer analysis were included in the inclusion list. The MS^2^ parameters were as follows: 70,000 resolution, 5 E5 AGC, 100-ms maximum injection time, 1.0 *m/z* isolation window, and 30 NCE. MetFrag *in silico* fragmentation prediction software was used to aid in metabolite identification (38). Target molecules were searched against KEGG and PubChem databases with a 5-ppm error. Candidate molecules from the databases were then processed against the MS/MS spectra of the target molecule with 5-ppm and 0.01-m/zabs settings. The top results of the *in silico* fragmentation were analyzed for putative identification. Putative identifications were then verified by comparing the experimental MS/MS to the MS/MS of the commercial standard.

### Accession number(s).

The metagenome sequences from this study are available under the BioProject identifier PRJNA491522 (https://www.ncbi.nlm.nih.gov/sra/PRJNA491522). The metabolomics data are available from the MetaboLights database under the accession number MTBLS753 (https://www.ebi.ac.uk/metabolights/MTBLS753).
