# Supplementary material for: Gut Microbial and Metabolic Responses to Salmonella enterica Serovar Typhimurium and Candida albicans
Source: mBio. 2018 Nov 6;9(6):e02032-18. doi: 10.1128/mBio.02032-18 (PMC6222126; doi:10.1128/mBio.02032-18)
Supplement: TABLE S1 [file mbo005184150st1.docx]

Supplemental Table 1. Biosynthetic gene clusters predicted in humanized microbiota

| Cluster Name | Count |
| --- | --- |
| putative | 486 |
| saccharide | 345 |
| fatty_acid | 117 |
| sactipeptide | 34 |
| fatty acid -saccharide | 22 |
| nrps | 19 |
| arylpolyene | 14 |
| thiopeptide | 12 |
| bacteriocin | 10 |
| siderophore | 4 |
| lantipeptide | 3 |
| other | 3 |
| resorcinol | 3 |
| terpene | 2 |
| hserlactone | 2 |
| sactipeptide-cf_saccharide | 1 |
| sactipeptide-nrps | 1 |
| bacteriocin-proteusin | 1 |
| sactipeptide-lantipeptide | 1 |
| t1pks-nrps | 1 |
