# Supplementary material for: Gut Microbial and Metabolic Responses to Salmonella enterica Serovar Typhimurium and Candida albicans
Source: mBio. 2018 Nov 6;9(6):e02032-18. doi: 10.1128/mBio.02032-18 (PMC6222126; doi:10.1128/mBio.02032-18)
Supplement: TABLE S2 [file mbo005184150st2.docx]

Supplemental Table 2. Features of interested in humanized infected mice

| **Molecular weight** | **Retention time (min)** | **charge** | **MS/MS Identification** | **Overabundant in *Salmonella* or *Candida*** |
| --- | --- | --- | --- | --- |
| 247.1417 | 1.391 | 1 | Hydroxybutyrylcarnitine | *Salmonella* |
| 247.1417 | 1.625 | 1 | Hydroxybutyrylcarnitine | *Salmonella* |
| 256.1401 | 0.799 | 1 |  | *Salmonella* |
| 261.0303 | 1.772 | 1 |  | *Salmonella* |
| 285.1143 | 1.002 | 1 |  | *Salmonella* |
| 336.0561 | 1.16 | 1 |  | *Salmonella* |
| 347.0626 | 1.052 | 1 |  | Both |
| 348.0467 | 1.08 | 1 | Inosine monophosphate | *Salmonella* |
| 363.0575 | 1.093 | 1 |  | *Salmonella* |
| 371.2516 | 13.487 | 1 |  | *Salmonella* |
| 426.0879 | 0.832 | 1 | Glutathione-cysteine disulfide | *Salmonella* |
| 483.1088 | 1.048 | 2 |  | *Salmonella* |
| 503.8396 | 0.741 | 1 |  | *Salmonella* |
| 508.3609 | 22.75 | 1 |  | *Salmonella* |
| 555.13 | 1.437 | 2 |  | *Salmonella* |
| 612.1513 | 1.789 | 1 | Glutathione disulfide | *Salmonella* |
| 635.3753 | 13.747 | 2 |  | *Salmonella* |
| 691.8275 | 0.733 | 1 |  | *Salmonella* |
| 701.492 | 22.63 | 1 |  | *Salmonella* |
| 726.3701 | 13.302 | 2 |  | *Salmonella* |
| 759.8148 | 0.731 | 1 |  | *Salmonella* |
| 837.8299 | 0.72 | 1 |  | *Salmonella* |
| 205.0773 | 1.597 | 1 |  | *Candida* |
| 263.0904 | 1.592 | 1 |  | *Candida* |
| 268.0518 | 9.114 | 1 |  | *Candida* |
| 336.1797 | 13.536 | 1 |  | *Candida* |
| 457.2576 | 14.042 | 1 |  | *Candida* |
| 487.2318 | 18.094 | 1 |  | *Candida* |
| 487.268 | 14.205 | 1 |  | *Candida* |
| 514.3227 | 11.698 | 2 |  | *Candida* |
| 577.2232 | 1.429 | 1 |  | *Candida* |
