# Supplementary material for: Gut Microbial and Metabolic Responses to Salmonella enterica Serovar Typhimurium and Candida albicans
Source: mBio. 2018 Nov 6;9(6):e02032-18. doi: 10.1128/mBio.02032-18 (PMC6222126; doi:10.1128/mBio.02032-18)
Supplement: TABLE S3 [file mbo005184150st3.docx]

Supplemental Table 3. Strains used in humanized community

| **Genus** | **Species** | **ATCC** | **DSMZ** | Also known as |
| --- | --- | --- | --- | --- |
| *Akkermansia* | *muciniphila* | BAA-835 | 22959 |  |
| *Alistipes* | *indistinctus* | NA | 22520 |  |
| *Anaerococcus* | *hydrogenalis* | 49630 | 7454 |  |
| *Anaerotruncus* | *colihominis* | na | 17241 |  |
| *Bacteroides* | *caccae* | 43185 | 19024 |  |
| *Bacteroides* | *cellulosilyticus* | na | 14838 |  |
| *Bacteroides* | *coprophilus* | na | 18228 |  |
| *Bacteroides* | *dorei* | na | 17855 |  |
| *Bacteroides* | *eggerthii* | 27754 | 20697 |  |
| *Bacteroides* | *finegoldii* | na | 17565 |  |
| *Bacteroides* | *intestinalis* | na | 17393 |  |
| *Bacteroides* | *ovatus* | 8483 | na |  |
| *Bacteroides* | *plebeius* | na | 17135 |  |
| *Bacteroides* | *stercoris* | 43183 | na |  |
| *Bacteroides* | *thetaiotaomicron3731* | na | na |  |
| *Bacteroides* | *thetaiotaomicron7330* | na | na |  |
| *Bacteroides* | *thetaiotaomicronVPI-5482* | 29148 | na |  |
| *Bacteroides* | *uniformis* | 8492 | na |  |
| *Bacteroides* | *vulgatus* | 8482 | na |  |
| *Bacteroides* | *WH2* | na | na | *Bacteroides thetaiotamicron, Bacteroides cellulolyticus* |
| *Bacteroides* | *xylanisolvens* | na | 18836 |  |
| *Bifidobacterium* | *adolescentis* | 15703 | na |  |
| *Bifidobacterium* | *angulatum* | 27535 | 20098 |  |
| *Bifidobacterium* | *bifidum* | 29521 | 20456 |  |
| *Bifidobacterium* | *dentium* | 27678 | na |  |
| *Bifidobacterium* | *pseudocatenulatum* | 27919 | 20438 |  |
| *Blautia* | *hansenii* | 27752 | 20583 |  |
| *Blautia* | *luti* | na | 14534 |  |
| *Catenibacterium* | *mitsuokai* | na | 15897 |  |
| *Citrobacter* | *youngae* | 29220 | na |  |
| *Clostridium* | *asparagiforme* | na | 15981 |  |
| *Clostridium* | *bartlettii* | na | 16795 | *Intestinibacter bartlettii* |
| *Clostridium* | *bolteae* | BAA-613 | 15670 |  |
| *Clostridium* | *hathewayi* | na | 13479 |  |
| *Clostridium* | *hiranonis* | na | 13275 |  |
| *Clostridium* | *hylemonae* | na | 15053 |  |
| *Clostridium* | *leptum* | 29065 | 753 |  |
| Clostridium | M62_1 | na | na |  |
| *Clostridium* | *nexile* | 27757 | 1787 |  |
| *Clostridium* | *nexile-related* | na | na | *Tyzzerella nexilis* |
| *Clostridium* | *ramosum* | 25582 | 1402 |  |
| *Clostridium* | *scindens* | 35704 | 5676 |  |
| *Clostridium* | *spiroforme* | 29900 | 1552 |  |
| *Clostridium* | *sporogenes* | 15579 | na |  |
| *Clostridium* | *symbiosum* | 14940 | 934 |  |
| *Collinsella* | *aerofaciens* | 25986 | 3979 |  |
| Collinsella | aerofaciens | 25986 | 3979 |  |
| *Collinsella* | *intestinalis* | na | 13280 |  |
| *Collinsella* | *stercoris* | na | 13279 |  |
| *Coprococcus* | *comes* | 27758 | na |  |
| *Coprococcus* | *eutactus* | 27759 | na |  |
| *Desulfovibrio* | *piger* | na | na | GOR1 |
| *Dorea* | *formicigenerans* | 27755 | 3992 |  |
| *Dorea* | *longicatena* | na | 13814 |  |
| *Edwardsiella* | *tarda* | 23685 | na |  |
| Edwardsiella | tarda | 23685 | na |  |
| *Enterobacter* | *cancerogenus* | 35316 | na |  |
| *Escherichia* | *coliK12* | na |  |  |
| *Escherichia* | *fergusonii* | 35469 | 13698 |  |
| *Eubacterium* | *biforme* | 27806 | 3989 |  |
| *Eubacterium* | *cylindroides* | na | na |  |
| *Eubacterium* | *dolichum* | 29143 | 3991 |  |
| *Eubacterium* | *eligens* | 27750 | 3376 |  |
| *Eubacterium* | *hallii* | 27751 | 3353 |  |
| *Eubacterium* | *plautii* | 29863 | na | *Clostridium orbscindens;  Flavonifractor plautii* |
| Eubacterium | rectale | 33656 |  |  |
| *Eubacterium* | *ventriosum* | 27560 | na |  |
| *Faecalibacterium* | *prausnitzii M21/2* | na | na |  |
| *Fusobacterium* | *varium* | na | na | JCM6320 |
| *Holdemania* | *filiformis* | 51649 | 12042 |  |
| *Lactobacillus* | *reuteri* | na | 20016 |  |
| *Lactobacillus* | *ruminis* | 27780 | 20403 |  |
| *Marvinbryantia* | *formatexigens* | na | 14469 |  |
| *Megamonas* | *funiformis* | na | 19343 |  |
| *Mitsuokella* | *multacida* | 27723 | 20544 |  |
| *Parabacteroides* | *distasonis* | 8503 | 20701 |  |
| *Parabacteroides* | *johnsonii* | na | 18315 |  |
| *Parabacteroides* | *merdae* | 43184 | 19495 |  |
| *Proteus* | *penneri* | 35198 | na |  |
| *Providencia* | *alcalifaciens* | na | na |  |
| *Providencia* | *rettgeri* | na | 1131 |  |
| *Providencia* | *rustigianii* | 33673 | 4541 |  |
| *Providencia* | *stuartii* | 25827 | na | *Clostridium* sp. GM2/1 |
| *Roseburia* | *intestinalis* | na | 14610 |  |
| *Ruminococcus* | *gnavus* | 29149 | na |  |
| *Ruminococcus* | *hydrogenotrophicus* | na | 10507 | *Blautia  hydrogenotrophicus* |
| *Ruminococcus* | *lactaris* | 29176 | na |  |
| *Ruminococcus* | *obeum* | na | na |  |
| *Ruminococcus* | *torques* | 27756 | na |  |
| *Streptococcus* | *infantarius* | BAA-102 | na |  |
| *Subdoligranulum* | *variabile* | na | 15176 |  |
| *Victivallis* | *vadensis* | BAA-548 | 14823 |  |
